# The Impact of Design Misspecifications on Survival Outcomes in Cancer Clinical Trials

**DOI:** 10.3390/cancers17162609

**Published:** 2025-08-08

**Authors:** Fang-Shu Ou, Tyler Zemla, Jennifer G. Le-Rademacher

**Affiliations:** Division of Clinical Trials and Biostatistics, Mayo Clinic, Rochester, MN 55905, USA; zemla.tyler@mayo.edu (T.Z.); le-rademacher.jennifer@mayo.edu (J.G.L.-R.)

**Keywords:** clinical trials, misspecification, survival endpoints

## Abstract

**Background/Objectives**: Results from a well-designed trial provide evidence to support approval of truly effective treatments or discontinuation of ineffective treatments. However, the information available at the time of trial design may be limited which may lead to underpowered trials. This work aims to evaluate the impact of design assumption misspecifications on the statistical power of randomized trials with survival outcomes. **Methods**: The impact of the design assumption misspecifications on statistical power of four different statistical methods was investigated in a simulation study. The methods include the log-rank test, MaxCombo test, the test of difference in survival probability, and test of difference in restricted mean survival time (RMST). The deviations considered include the survival rate in the control arm, the expected treatment effect in terms of magnitude and pattern, accrual rate, and drop-out rate. **Results**: Deviations in the control arm’s survival distribution have no impact on the power of the log-rank and MaxCombo tests but it affects the trial duration since trials designed with these tests require the total number of events to be met before the final analysis can be conducted. Misspecified treatment effect has similar effect on the statistical power of all four methods. When the proportional hazards assumption is misspecified, the RMST is more robust with a larger early treatment effect, while the survival probability and the MaxCombo tests are more robust with a larger late treatment effect and crossing hazards. **Conclusions**: Selecting the appropriate statistical tests to design a trial depends on the goal of the trial, the mechanism of action of the experimental treatment, the survival quantity of clinical interest, and the pattern of the expected treatment effect. The final design should be based on assumptions that are as accurate as possible, and the potential impacts of deviations from these assumptions on the trial’s statistical power should be carefully considered.

## Simple Summary

Clinical trial design relies on assumptions that may change over time, potentially affecting the accuracy of results. This study evaluates how deviations in design assumptions impact the statistical power of randomized trials with survival endpoints. Findings show that incorrect assumptions can significantly affect all methods similarly. Thus, it is crucial to base trial designs on the most accurate assumptions and consider potential impacts on statistical power.

## 1. Introduction

Regulatory approval of a cancer drug relies on the demonstration of its benefit compared to a control (whether a placebo or a current standard of care), typically in randomized clinical trials with a time-to-event endpoint, such as overall survival, progression-free survival, and disease-free survival [1].

Results from a well-designed trial—with the optimal survival benefit selected to reflect the mechanism of action of the experimental treatment, the design assumptions well-justified and reflective of the target patient population, as well as realistic expectations of the treatment effect—provide evidence to support approval of truly effective treatments or discontinuation of ineffective treatments. However, the information available at the time of trial design may be limited and change overtime, resulting in a trial whose design does not fully capture the effect of the new treatment or based on assumptions that deviate from the expected outcome which may lead to erroneous conclusions.

A deviation that has been noted in the past and has received more attention in recent years is the proportional hazards assumption. The log-rank test, which is often the basis for sample size estimation in phase III oncology trial, is the most powerful test when the hazard ratio between the new treatment and the control treatment is constant over time, known as proportional hazards [2]. However, Trinquart et al. [3] reported that 13 out of 54 (24%) published trials failed a formal test of proportional hazards assumptions. Even when a test of proportional hazard does not show statistically significant violation, the treatment effect can still vary over time and impact the trial outcome in a meaningful way [4,5]. With the recent surge of approvals of immune-oncology therapy, the proportional hazards assumption has become more questionable. Results of recent immuno-oncology trials indicate that the effects of immunotherapy are often delayed and the assumption of proportional hazards do not hold in immuno-oncology trials [6]. For example the KEYNOTE-045 trial [7] showed crossing survival curves (for both overall and progression-free), and the CheckMate 238 trial [8] showed late separation where there was no survival difference in the first 3 months.

In addition to the deviation in proportional hazard assumption, other deviations in trial design assumptions are possible. Yet design assumptions and the impact of their deviations from the observed data are rarely evaluated at trial completion or discussed in trial publications [9]. The deviation of baseline hazard in the control arm can occur when the survival rates of the control arms initially assumed during trial design do not accurately reflect the improvement in survival years later due to new scientific development or changes in standard of care. For example, the KEYNOTE-189 trial [10] was designed assuming the median overall survival for patients receiving chemotherapy alone as the first-line treatment for metastatic non-small-cell lung cancer was 13 months, however the observed median overall survival was 11.3 months; median progression-free survival assumed in the design was 6.5 months compared with the observed median of 4.8 months. This is an example where the observed survival experience in the control arm was worse than that was assumed in the design. Whereas the CheckMate 067 trial [11] is an example where the observed survival experience in the control arm was better than the design assumption. Specifically, the observed 24-month overall survival probability for patients receiving ipilimumab as the first-line treatment for advanced melanoma was 45% compared to the 24.4% assumed for the design.

Other deviations, such as deviations in enrollment rate and drop-out rate, have also been seen in clinical trials. For example, CALGB/SWOG 80702 (Alliance) planned to finish enrollment in 3.125 years but it took more than 4 years (June 2010 to November 2015) to reach full enrollment [12]. The PROSPECT trial started with a sample size of 1016 patients and subsequently increased the target enrollment to 1120 due to a higher than anticipated drop-out post-randomization [13]; it reached full enrollment with 1194 patients [14]. The prevalence of these type of deviations is difficult to gauge because the information is often omitted from the clinical trial manuscripts [15].

The aim of this work is not to compare the performance of various survival analysis methods, which have been well-published. Our aim is to evaluate how deviations in design assumptions affect statistical power (and Type I error when appropriate) for each method, while also accounting for the follow-up scheme associated with the method. Specifically, we consider the following deviations: (1) survival distribution of the control arm (referred to as baseline survival), (2) expected treatment effect (pattern and magnitude), (3) accrual rate, and (4) drop-out rate. In addition to evaluate the impact of deviations for the most common method, log-rank test, we also include other statistical tests, such as the difference in survival probabilities at a prespecified time point, the restricted mean survival time (RMST), and the MaxCombo tests, in our investigations.

The rest of the manuscript is organized as follows. In Section 2, we define the different test statistics used and describe their associated design, as well as detailing the simulation setup. The study power and Type I error from each test/deviation combination are described in Section 3. Section 4 and Section 5 consist of the conclusion and discussions, respectively.

## 2. Methods

For simplicity, this exposition describes a two-arm randomized trial with a 1:1 randomization ratio. However, all concepts can be generalized to trials with multiple arms and to other randomization ratios.

### 2.1. Design Framework

Let X be the time to the event of interest. Let S(x)=Pr(X>x) denote the survival function, the probability of an individual surviving beyond time x, and the hazard function be defined as $h\left(x\right)=\;\underset{\Delta x\to 0}{lim}\;\frac{Pr(x\leq X<x+\Delta x|X\geq x)}{\Delta x}$. Let $S_{C}(x)$ and $h_{C}(x)$ be the survival function and hazard function for the control arm, respectively. Similarly, let $S_{T}(x)$ and $h_{T}(x)$ be the survival function and hazard function for the treatment arm, respectively. Below are the statistical hypotheses being evaluated in this paper, grouped by associated design.

#### 2.1.1. Event-Based Design

The log-rank test and the MaxCombo test are methods to evaluate the hazard rate, specifically testing the following hypotheses:

$$H_{O}:h_{C}\left(x\right)=h_{T}\left(x\right)\;\mathrm{for}\;\mathrm{all}\;x\leq T\;\mathrm{versus}\;H_{1}:h_{C}\left(x\right)\neq h_{T}\left(x\right)\;\mathrm{for}\;\mathrm{some}\;x\leq T$$

where T is the largest time at which both arms have at least one subject at risk. While the log-rank test statistic gives equal weight to differences in the observed and expected mortality over time, the MaxCombo test statistic is based on the maximum value of a combination of tests with varying weights (some give more weight to earlier differences and others give more weight to later differences). For the MaxCombo tests, it is necessary to specify the weight function and the components a priori.

In trials designed based on log-rank test and MaxCombo test, the size of a trial is stated in terms of the total number of events of interest. Under this type of design, all patients are followed until the time when the prespecified total number of events are observed, regardless of when they are enrolled on the trial. Therefore, follow-up duration of patients within the same trial can vary widely, with shorter duration for more recent enrollment and longer duration for earlier enrollment.

#### 2.1.2. Fixed Follow-Up Duration Design

In contrast to the log-rank test and the MaxCombo test, which are based on the hazard rate, the other two methods are based on the survival probability. The hypotheses for testing the difference in survival probability at a prespecified time t are:

$$H_{O}:S_{C}\left(t\right)=S_{T}\left(t\right)\;\mathrm{versus}\;H_{1}:S_{C}\left(t\right)\neq S_{T}\left(t\right)$$

where t is the prespecified time of the survival probability of interest and the hypotheses for testing the difference in the RMST at time τ are

$$H_{O}:\mu_{C}(\tau )=\mu_{T}(\tau )\;\mathrm{versus}\;H_{1}:\mu_{C}(\tau )\neq \mu_{T}(\tau )$$

where $\mu \left(\tau \right)=\int_{0}^{\tau }S\left(x\right)dx$ and τ is the prespecified restriction time. Although it is not within the scope of this work to discuss how to select t and τ, it is important to note that their selection should be based on the time point that is most clinically relevant for the trial being designed and that the statistical power of the survival probability test and the RMST test depend on time t and τ, respectively.

In trials designed based on survival probability and RMST, the trial size is stated in terms of the total number of patients enrolled. In both methods, survival experience beyond the prespecified time of interest (time t for survival probability test and τ for RMST test) does not contribute to the hypothesis test. Under this type of design, patients are enrolled and followed until they experience the event of interest or reach the prespecified time, t and τ, whichever occurs first.

**Figure 1:**
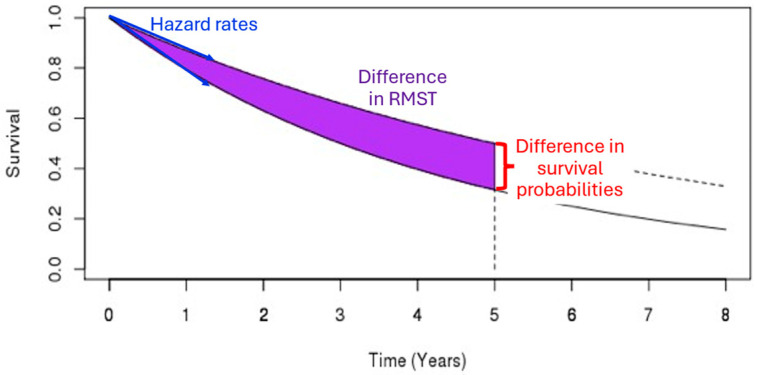
The survival quantity being compared for the different methods. The log-rank test and the MaxCombo test compare the hazard rate (a function of the slope of the survival curves, indicated by the blue arrows), the difference in survival probabilities at t = 5 years (distance denoted by the red bracket), and the difference in mean survival times restricted at τ = 5 years (the difference between the areas under the survival curves, purple shaded area).

Table 1 summarizes the design framework, and Figure 1 illustrates the quantities being compared for the different endpoints.

### 2.2. Simulation Setup

A simulation study was conducted to evaluate the impact of deviations on the statistical power and Type I error (when appropriate).

#### 2.2.1. Original Designs

The trial design that will be used as the reference for all comparisons in this paper is based on the following assumptions:

- Two arms randomized at a 1:1 ratio;
- Survival distribution of control arm:
  ○ follows exponential distribution;
  ○ median survival time = 3 years;
- Treatment effect:
  ○ proportional hazards;
  ○ hazard ratio (HR, experiment versus control) = 0.6;
- Type I error of one-sided 0.025 and 90% power;
- Accrual rate = 10 pts/month, uniformly distributed.

The resulting design based on the log-rank test requires a total of 162 events. With the assumed accrual rate, the trial needs to enroll a total of 350 patients (175 per arm) with an anticipated total trial duration of 5 years, defined as the time from first patient enrollment to statistical analysis of the primary endpoint. This design provides the same power for the MaxCombo tests. For this work, we focus on 2 MaxCombo tests: a 2-component with weights [(0,0) and (0,0.5)] and a 3-component with weights [(0,0), (0,0.5), and (0.5,0.5)] which are recommended for oncology trials from previously published work [16]. More details on MaxCombo tests are provided in the Appendix A.

Given that the goal of this work is to show how the deviations from the original design assumptions affect the statistical power within each of the different methods and not based on specific clinical setting, we selected t and τ so that the original designs for the fixed follow-up duration designs achieve as close to 90% power as possible based on the same assumptions as the original log-rank based design. This leads to the selection of t=3.5 years (empirical power 89.06%) for the survival probability test and τ=4.5 years (empirical power 90.36%) for the RMST test.

#### 2.2.2. Deviations Evaluated

**Figure 2:**
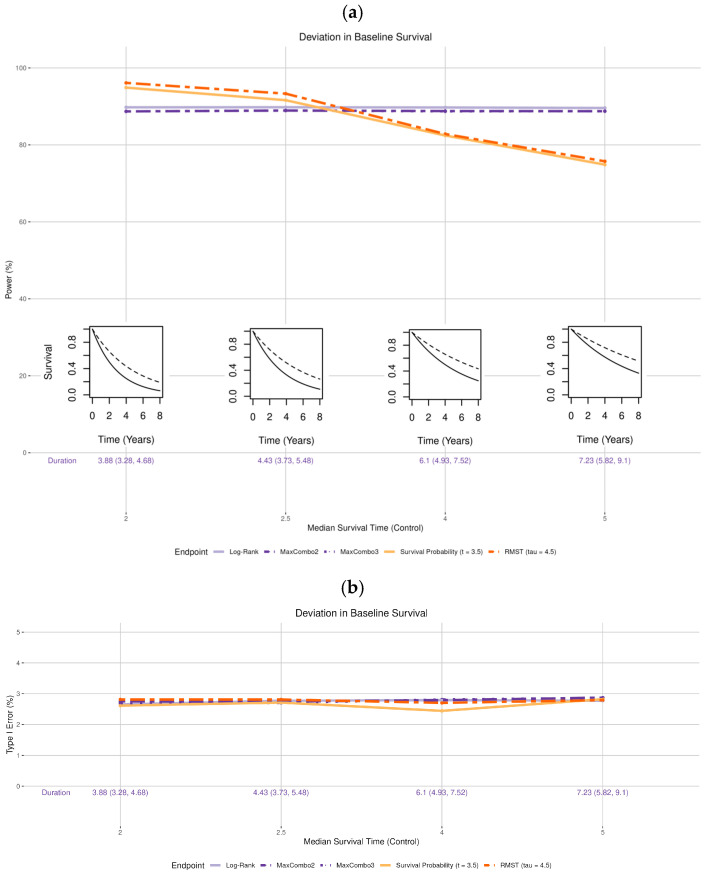
Changes in (**a**) statistical power and (**b**) Type I error associated with misspecified survival distribution of the control arm. The insets in (**a**) show four of the simulation scenarios where the control arm is drawn with solid black lines and the experimental arm is drawn with dashed black lines.

**Figure 3:**
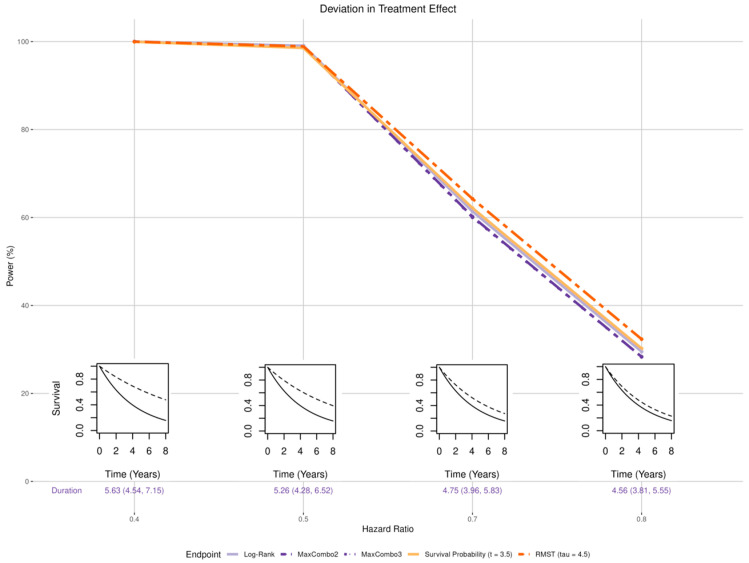
Changes in statistical power associated with misspecified magnitude of treatment effect. The insets show four of the simulation scenarios where the control arm is drawn with solid black lines and the experimental arm is drawn with dashed black lines.

**Figure 4:**
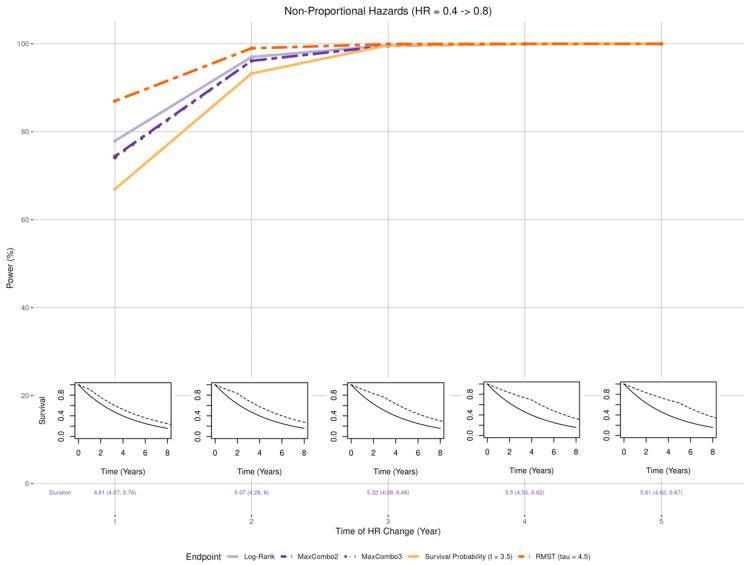
Changes in statistical power associated with misspecified treatment effect, non-proportional hazards with early benefit. The insets show five of the simulation scenarios where the control arm is drawn with solid black lines and the experimental arm is drawn with dashed black lines.

**Figure 5:**
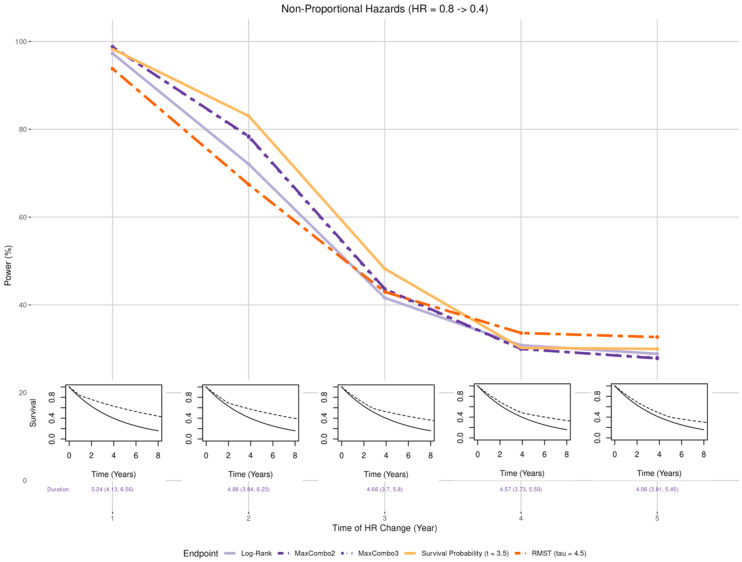
Changes in statistical power associated with misspecified treatment effect, non-proportional hazards with late benefit. The insets show five of the simulation scenarios where the control arm is drawn with solid black lines and the experimental arm is drawn with dashed black lines.

**Figure 6:**
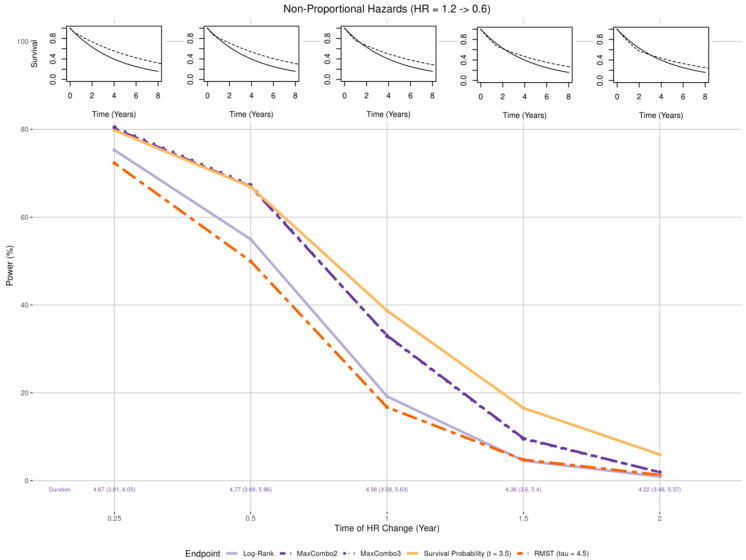
Changes in statistical power associated with misspecified treatment effect, non-proportional hazards with crossing hazards. The insets show five of the simulation scenarios where the control arm is drawn with solid black lines and the experimental arm is drawn with dashed black lines.

The following deviations from the original design were considered:

1. Survival distribution of the control arm (Figure 2 insets): the observed median survival was set to be shorter than the expected time of 3 years (2 and 2.5 years, i.e., worse survival) and longer than expected (4 and 5 years, i.e., better survival).
2. Treatment effect:
  - Magnitude (Figure 3 insets): the treatment effect was set to be larger than the expected hazard ratio of 0.6 (HR of 0.4 and 0.5) and smaller than expected (HR of 0.7 and 0.8).
  - Non-proportional hazards (NPH):
    - Early benefit (Figure 4 insets): Larger than expected early treatment effect (HR = 0.4) in the first k years post enrollment and smaller than expected effect (HR = 0.8) after k years where k = 1, 2, 3, 4, and 5;
    - Late benefit (Figure 5 insets): Smaller than expected early treatment effect (HR = 0.8) in the first k years post enrollment and larger than expected effect (HR = 0.4) after k years where k = 1, 2, 3, 4, and 5;
    - Crossing hazard (Figure 6 insets): Worse than expected survival in treatment arm (HR = 1.2) in the first k years post enrollment and the same as expected treatment effect (HR = 0.6) after k years where k = 0.25, 0.5, 1, 1.5, and 2.
3. Accrual rate: Faster than expected accrual (i.e., full enrollment in 2 and 2.5 years) and slower than expected accrual (i.e., full enrollment in 3.5 and 4 years).
4. Drop-out rate: The original design assumes no drop-out. We incorporated various drop-out rates. The drop-out process is assumed to follow the exponential distribution with 15%, 30%, 45%, and 60% cumulative proportion by 5 years, independent of the survival process (i.e., non-informative censoring). Note that the observed drop-out rates vary based on the time of statistical analysis and are not the same as the cumulative 5-year rate. For example, for the 30% drop-out setting in simulation, the median observed drop-out rates by 3.5 and 4.5 years were 16.6% and 19.2%, respectively; for the 60% drop-out setting, the median observed drop-out rates by 3.5 and 4.5 years were 36.3% and 40.6%, respectively. Additionally, we did not incorporate informative censoring, where the censoring process and the survival process are dependent, as it would not only affect statistical power but also raise concerns about the validity of the trial. Addressing this issue is beyond the scope of the current manuscript.

All deviations were evaluated for their impact on the statistical power under each of the four statistical methods with corresponding follow-up schemes as described in Section 2.1. Type I error was also evaluated for deviations in survival distribution of the control arm, the accrual rate, and the drop-out rate.

Simulations were performed for each scenario with 10,000 iterations. The power was calculated as the percent of tests (out of 10,000) rejecting the null hypothesis (under alternative). The Type I error was calculated as the percent of tests (out of 10,000) rejecting the null hypothesis (under null). The simulation was performed using the R software version 4.4.1 [17]. The MaxCombo tests and RMST tests were performed using nph package version 2.1 [18] and survRM2 package version 1.0-4 [19], respectively, in R.

## 3. Results

### 3.1. Deviation in Survival Distribution of the Control Arm

The log-rank, MaxCombo2, and MaxCombo3 tests maintained 90% power regardless of the median survival time of the control arm (Figure 2a). However, it is important to note that the trial duration is highly dependent on the baseline survival distribution (noted at the bottom of Figure 2). If the true median survival time for the control arm is 2 years (versus 3 years as assumed in the original design), the trial duration is much shorter (median trial duration: 3.88 years; range: 3.28–4.68 years) than the expected duration of 5 years in the original design; whereas if the true control arm median survival time is 5 years, the trial duration can take much longer (median trial duration: 7.23 years; range: 5.82–9.1 years).

On the other hand, the power of survival probability and RMST are affected by the deviation in the survival distribution in the control arm. When survival in the control arm is worse (median 2 or 2.5 years) than the original design assumption (median 3 years), the power for both survival probability and RMST are higher than the 90%. When the baseline survival is better than the original design assumption (median 4 or 5 years), the power for both tests decrease.

Type I error maintains stable for all tests regardless of the deviation (Figure 2b).

### 3.2. Deviation in the Treatment Effect

#### 3.2.1. Magnitude of Effect

Misspecified treatment effect size has similar impact on all five tests (Figure 3). A larger treatment effect (HR = 0.4 or 0.5) resulted in higher statistical power and a smaller treatment effect (HR = 0.7 or 0.8) resulted in lower power. The power loss is not substantially different across different tests. For log-rank, MaxCombo2, and MaxCombo3 tests which rely on event-driven follow-up, a larger treatment effect (HR = 0.4 and 0.5) resulted in a slightly extended trial duration (median trial duration of 5.63 and 5.26 years, respectively) a smaller treatment effect (HR = 0.7 and 0.8) resulted in a slightly shortened trial duration (median trial duration of 4.75 and 4.56 years, respectively).

#### 3.2.2. Non-Proportional Hazards, Larger Early Benefit

Overall pattern of change in statistical power is similar across all five tests when the proportional hazards assumption was violated. Figure 4 shows that when there is a larger early effect (HR = 0.4) and a smaller late effect (HR = 0.8), the power for all five tests increased as the time of the effect change (*k*) goes from 1 year to 5 years (corresponding to longer duration with larger treatment effect). When the HR changed from 0.4 to 0.8 at 1 or 2 years (i.e., *k* = 1, 2) the statistical power of the RMST test is the least affected while survival probably test is the most affected. When the HR change occurred at 4 years or later (i.e., *k* = 4, 5), all five tests reached the maximum statistical power of 100%. For log-rank, MaxCombo2, and MaxCombo3 test which rely on event-driven follow-up, the early change in HR (i.e., *k* = 1) slightly reduce the trial duration (median trial duration = 4.81 years) and a later change slightly prolong the trial duration (median trial duration = 5.61 years).

#### 3.2.3. Non-Proportional Hazards, Larger Late Benefit

Figure 5 shows that when there is a smaller early effect (HR = 0.8) and a larger late effect (HR = 0.4), the power for all tests decreased as the time of the HR change (*k*) goes from 1 year (corresponding to shorter duration with smaller treatment effect) to 5 years (corresponding to longer duration with smaller treatment effect) with the survival probability less affected than others when the larger benefit occurred before 3.5 years; whereas the RMST test is more affected than other tests when the HR change occurred before 3.5 years but becomes less affected when the change occurred after 3.5 years. For log-rank, MaxCombo2, and MaxCombo3 test which rely on event-driven follow-up, the early change in HR (i.e., *k* = 1) slightly prolongs the trial duration (median trial duration = 5.24 years) and a later change slightly reduces the trial duration (median trial duration = 4.56 years).

#### 3.2.4. Non-Proportional Hazards, Crossing Hazard

A more extreme case of non-proportional hazards is where the new treatment is associated with worse survival in the early period and better survival in the late period. Figure 6 shows the statistical power when the HR = 1.2 before time *k* and HR = 0.6 after time *k*. The statistical power of all five tests decreased as the time of change (*k*) goes from 3 months to 2 years. Of note, in this scenario, the statistical power of the survival probability test is the least affected whereas the RMST test is the most affected. The statistical power of the MaxCombo tests is less affected than the that of the log-rank test in this scenario. Due to the faster rate of events early on, the trial duration is reduced for tests rely on event-driven follow-up (median trial duration between 4.22 and 4.87 years).

### 3.3. Deviation in the Accrual Rate

The deviation in accrual rate does not impact the statistical power nor the Type I error when sufficient follow-up is performed, i.e., patients are followed until pre-specified number of events for log-rank, MaxCombo2, and MaxCombo3 tests and followed until pre-specified duration of follow-up for survival probability and RMST. However, the accrual rate does impact the total trial duration for all tests. For tests under event-driven follow-up scheme, a faster accrual, 2 years instead of 3, resulted in a shorter total trial duration (median: 4.44 years, range: 3.58–5.22 years to reach 162 events) and slower accrual, 4 years instead of 3, resulted in a longer trial duration (median: 5.54 years, range: 4.66–6.83 years to reach 162 events). For survival probability and RMST, the total trial duration is approximately the accrual duration plus the fixed-duration follow-up; therefore, the total trial duration is directly impacted by the accrual rate.

### 3.4. Deviation in the Drop-Out Rate

**Figure 7:**
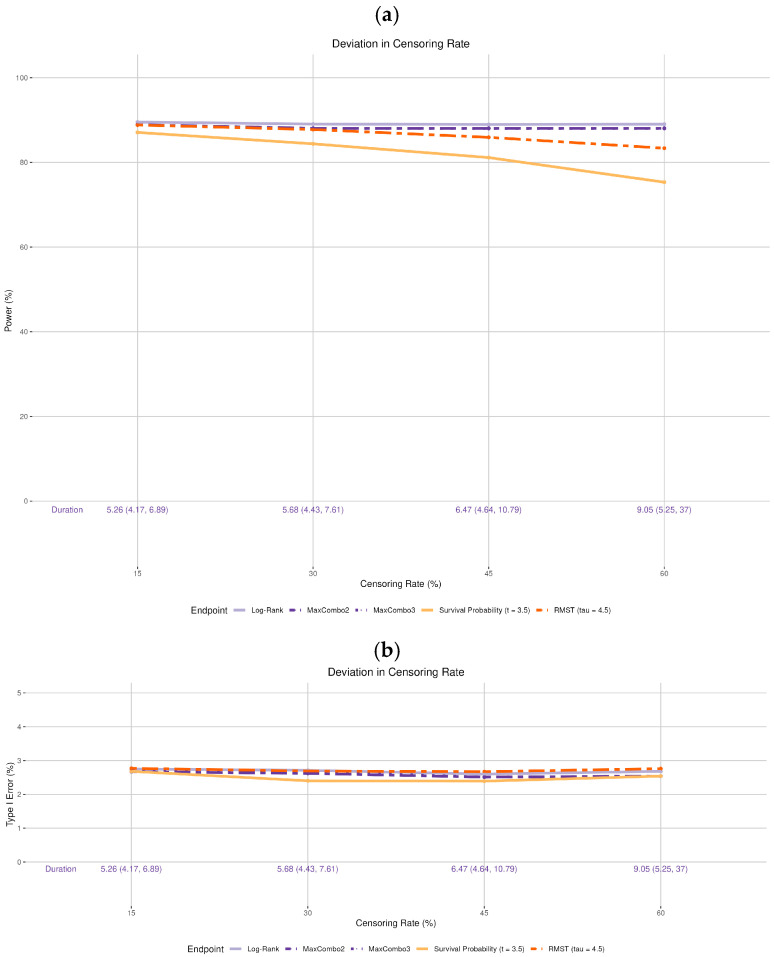
Changes in (**a**) statistical power and (**b**) Type I error associated with increased drop-out rate.

Drop-out rate has minimal impact on the statistical power of the log-rank and the MaxCombo tests (Figure 7a). This pattern is expected given that the power of the log-rank and the MaxCombo tests depend on the number of events, and by design the analysis is conducted when the number of events is reached. However, when the drop-out rate is very high, e.g., in the 60% drop-out scenario, the total number of events required were never reached for 11% of the simulations, where the statistical analysis was conducted with less than 162 events, which slightly reduced the statistical power. It is important to note that, although the power was less impacted for event-driven tests, the trial duration can prolong dramatically, e.g., in the 60% drop-out scenario, the median study duration is around 9 years rather than the expected study duration of 5 years. In contrast, the impact of drop-out on statistical power is more pronounced for the survival probability and the RMST tests, due to more patients being censored at the pre-specified analysis time. However, the trial duration based on these methods is not affected since patients are followed for a fixed duration.

Drop-out rate has minimal impact on the Type I error (Figure 7b).

## 4. Discussion

This paper evaluated the impact of design misspecifications on the statistical power and Type I error on each of the 4 statistical methods that can be used to design clinical trials with survival outcomes. The methods include the log-rank test and MaxCombo tests for comparing the hazard rate, the test comparing the difference in the survival probability at a fixed time, and the test comparing the difference in the restricted mean survival time. In all parameters examined, misspecifications have similar impact in terms of direction and magnitude of the statistical power in all methods. The impact to Type I error is negligible.

Some notable observations include:

1. Deviation in the control arm’s survival rate does not affect the power of the log-rank and MaxCombo but it affects the total duration of the trial. The power of the RMST test and survival probability decrease when the control arm’s survival is better than the assumed distribution and increase when the control arm survival is worse than the assumed rate. These changes in power are due to an interplay between the change in the magnitude of the survival difference (in both survival probability and RMST, resulting from the deviation from baseline survival rate) coupled with the change in the estimation precision due to the censoring rate at time t and τ. This observation is consistent with the results found in Appendix 4 of Eaton et al. for RMST [20].
2. When the proportional hazards assumption is misspecified, the RMST test is least affected when there is a larger early treatment effect; the survival probability was least affected when there is larger late treatment effect, especially when the larger treatment effect occurs prior to the prespecified time of the survival probability; while the survival probability test and MaxCombo were the least affected with crossing hazards. Of note, in scenarios where the statistical power of the survival probability test is less affected than the other tests, it is due to the fact that the hazard-based tests and the RMST test evaluate the cumulative effect of treatment where small treatment effect in the early period dilutes the overall effect and, similarly, the harm of the treatment in the early period cancels out benefit of the late period. In contrast, the survival probability only evaluates the survival difference at 3.5 years regardless of the direction of early treatment effect. The power of the MaxCombo tests is less affected than the log-rank and the RMST in scenarios with crossing hazard since the method is designed to select the weight combination that maximizes the difference.
3. While deviations in the drop-out rate and accrual rate are seldom discussed in clinical trial manuscripts, they can significantly prolong the trial duration. An excessively prolonged trial can dramatically increase the monetary cost of the trial, and the standard of care may change during the trial period, rendering the trial conclusions less relevant. Additionally, a high drop-out rate can also reduce the study power.

It is well known that the magnitude of the treatment effect strongly impacts the trial statistical power. Advances in immuno-oncology in recent years have led to a heightened interest in understanding the impact of non-proportionality on the log-rank test and alternative statistical methods that are not constrained by the proportional hazards assumption. The survival probability at a fixed time and the restricted mean survival time were alternative endpoints that do not require the proportional hazards assumption, while the MaxCombo test is a method that allows flexible weighing schemes to focus on time period with the largest difference in survival between the two treatment arms. The survival probability at a fixed time and the restricted mean survival time also provide intuitive interpretation under nonproportionality. Of these alternatives, the survival probability at a fixed time is the appropriate endpoint if the goal of the trial is to show that the new treatment increases the likelihood of being alive at that time point, regardless of the shape of the hazard leading up to that time point. The restricted mean survival time, on the other hand, uses cumulative data up to the restriction time and the restriction time can be selected so that the power of the RMST test can approach that of the log-rank test. MaxCombo test, depending on the components and weights chosen, can be more robust against different type of deviations compared to the log-rank test.

Misspecification in other parameters such as the control arm survival distribution has received little attention in the clinical trial literature. Our simulation study shows that deviation in the control arm survival distribution can have a moderate impact on the statistical power for survival probability test and restricted mean survival time endpoints. Although in trials designed with the log-rank test and MaxCombo test where all patients are followed until a fixed number of events are reached, this deviation has no impact on the power of the log-rank test but the total trial duration can be highly contracted or extended, in some cases, trials may be extended for years to reach the few final events. An advantage of trials designed with survival probability or restricted mean survival time is that the trial duration is more predictable. Patients are followed to time *t* for the survival probability and to time τ for the restricted mean survival time. Any follow-up beyond the prespecified time point does not contribute to the test statistics. A design with fixed follow-up duration is attractive for logistical reasons, for example, the time point of the final data analysis is known in advance so that resource allocation can be planned ahead of time. This design is also appropriate for trials with funding restricted to a fixed period. For designs using fixed follow-up duration, slower accrual rate directly affects the total trial duration which includes the accrual duration plus the fixed follow-up duration.

This manuscript uses simulations to evaluate the impact of deviations, which have inherent limitations. The simulations rely on specific parameter settings that may not fully capture the complexity of real-world data. We have chosen the parameter values to demonstrate the effect of deviations, but certainly, other parameter values are possible to represent the same deviation.

## 5. Conclusions

Selection of the appropriate quantity and associated statistical test to evaluate the survival benefit of a new treatment depends on multiple factors including the goal of trial, the mechanism of action of the experimental treatment, the survival quantity of clinical interest, and the pattern of the expected treatment effect. Although there are minor differences in how much misspecified assumptions affect the statistical power of the different statistical methods used, the overall pattern and impact of the mis-specified assumptions evaluated in this work seem to affect all methods considered in similar manner and the impact can be significant. Therefore, regardless of which method is used, the trial design should be based on as accurate assumptions as possible and potential impacts of deviations from these assumptions on the trial statistical power should be carefully considered.

## Figures and Tables

**Table 1:** Design framework for randomized trials with survival outcomes.

	Hazard Rate h(x)		Survival Probability S(t)	Restricted Mean Survival Time μ(τ)
Statistical Test	Log-rank test	MaxCombo test	Test of difference	Test of difference
Treatment effect quantified by	Hazard ratio	No corresponding quantity	Difference in survival probability	Difference in mean survival time
Trial size stated in terms of	The total number of events	The total number of events	Total number of patients enrolled	Total number of patients enrolled
Follow-up	All patients are followed until the total number of events are reached	All patients are followed until the total number of events are reached	Each patient is followed until event or time t whichever occurs first; follow-up beyond t does not contribute to test statistic	Each patient is followed until event or time τ whichever occurs first; follow-up beyond τ does not contribute to test statistic
Advantages	Uses all available data during follow-up; has the highest statistical power under proportional hazards assumption	Does not require proportional hazards assumption; uses all available data during follow-up	Does not require proportional hazards assumption; trial duration more predictable (depends only on the enrollment rate)	Does not require proportional hazards assumption; trial duration more predictable (depends only on the enrollment rate)
Disadvantages	Requires proportional hazards assumption (for optimal power and interpretability of hazard ratio); trial duration can be unpredictable (depends on time to reach number of events required)	Trial duration can be unpredictable (depends on time to reach number of events required)	Use only data up to time t	Use only data up to time τ

## Data Availability

R code is available upon request.

## Abbreviations

The following abbreviations are used in this manuscript:

RMST	Restricted mean survival time

## Appendix A

### MaxCombo Test

MaxCombo test is a combination test based on the weighted log-rank test using the Fleming-Harrington weight family $G^{\rho ,\gamma }$ [2].

Weighted log-rank test using the Fleming-Harrington weight family $G^{\rho ,\gamma }\;$ has the test statistics:

$$\chi^{2}=\frac{{\left[\sum_{t=1}^{D}w_{t}\left(o_{t}-e_{t}\right)\right]}^{2}}{\sum_{t=1}^{D}w_{t}^{2}\left(v_{t}\right)}$$

where

$o_{t}=d_{1t}$, observed number of events in group 1 at time t,
$e_{t}=n_{1t}\left(\frac{d_{t}}{n_{t}}\right)$, expected number of events in group 1 at time t,
$v_{t}=d_{t}\;n_{1t}\left(\frac{n_{t}-n_{1t}}{n_{t}^{2}}\right)\left(\frac{n_{t}-d_{t}}{n_{t}-1}\right)$, variance of expected number of events in group 1 at time t,
$d_{t}$ is the total number of events at time t,
$n_{t}$ is the total number of events at time t, for each event time t=1, ⋯, D,
$w_{t}={\left\{\;\hat{S}(t-)\right\}}^{\rho }\{1-{\;\hat{S}(t-)\}}^{\gamma }\;,\;\rho \geq 0,\;\gamma \geq 0$.

For example, considering ρ=0, 1  and γ=0, 1 as shown in the table below.

(ρ,γ)	$w_{t}$	**Type of Test**
(0, 0)	1	Log-rank
(1, 0)	$\left\{\;\hat{S}(t-)\right\}$	Test early difference
(0, 1)	$\;\{1-\;\hat{S}(t-)\}$	Test late difference
(1, 1)	$\left\{\;\hat{S}(t-)\}\{1-\;\hat{S}(t-)\right\}$	Test middle difference

MaxCombo test was proposed by the working group with pharmaceutical companies initiated by the U.S. Food & Drug Administration [6]. The test statistics was proposed to maximum combination (MaxCombo) using the Fleming-Harrington $G^{\rho ,\gamma }$ weight family $G^{\rho ,\gamma }$, ρ, γ≥0 which has the form:

$Z_{max}=max_{\rho ,\gamma }\{G^{0,0},G^{0,1},G^{1,0},G^{1,1}\}$.

In this work, we considered the following MaxCombo tests.

- Two-component MaxCombo test with weights [(0,0) and (0,0.5)], defined as $Z_{2,\;0.5}=max_{\rho ,\gamma }\{G^{0,0},G^{0,0.5}\}$.
- Three-component MaxCombo test with weights [(0,0), (0,0.5), and (0.5, 0)], defined as $Z_{3,\;0.5}=max_{\rho ,\gamma }\{G^{0,0},G^{0,0.5},G^{0.5,0}\}$.

We choose to use these combinations because these were recommended by the more recent research [16].
